# Molecular determinants of plaque size as an indicator of dengue virus attenuation

**DOI:** 10.1038/srep26100

**Published:** 2016-05-17

**Authors:** Kenneth Choon Meng Goh, Choon Kit Tang, Diana Catherine Norton, Esther Shuyi Gan, Hwee Cheng Tan, Bo Sun, Ayesa Syenina, Amjad Yousuf, Xin Mei Ong, Uma Sangumathi Kamaraj, Yin Bun Cheung, Duane J Gubler, Andrew Davidson, Ashley Lauren St John, October Michael Sessions, Eng Eong Ooi

**Affiliations:** 1 Duke-NUS Medical School, Singapore 169857, Singapore; 2 Duke University School of Medicine, Durham, North Carolina 27710, USA; 3 University of Bristol, Bristol BS8 1TD, UK

## Abstract

The development of live viral vaccines relies on empirically derived phenotypic criteria, especially small plaque sizes, to indicate attenuation. However, while some candidate vaccines successfully translated into licensed applications, others have failed safety trials, placing vaccine development on a hit-or-miss trajectory. We examined the determinants of small plaque phenotype in two dengue virus (DENV) vaccine candidates, DENV-3 PGMK30FRhL3, which produced acute febrile illness in vaccine recipients, and DENV-2 PDK53, which has a good clinical safety profile. The reasons behind the failure of PGMK30FRhL3 during phase 1 clinical trial, despite meeting the empirically derived criteria of attenuation, have never been systematically investigated. Using *in vitro, in vivo* and functional genomics approaches, we examined infections by the vaccine and wild-type DENVs, in order to ascertain the different determinants of plaque size. We show that PGMK30FRhL3 produces small plaques on BHK-21 cells due to its slow *in vitro* growth rate. In contrast, PDK53 replicates rapidly, but is unable to evade antiviral responses that constrain its spread hence also giving rise to small plaques. Therefore, at least two different molecular mechanisms govern the plaque phenotype; determining which mechanism operates to constrain plaque size may be more informative on the safety of live-attenuated vaccines.

The *in vitro* replication rate of pathogens is a common measure of fitness. A rapidly replicating virus in culture is interpreted as having greater fitness than slower growing strains^1^,^2^,^3^. Correspondingly, the size of plaques, which are caused by necrosis or apoptosis upon infection of a cell monolayer, is a common proxy measure of viral fitness. This empirically derived measurement of fitness is used in the development of live attenuated vaccines (LAVs), where strains that produce small plaques are repeatedly selected and purified for clinical development^1^,^4^,^5^.

While this empirical approach in vaccine development has yielded successful vaccines including measles^6^, mumps^7^ and yellow fever^8^,^9^, it has also led to failed clinical trials, where candidate vaccines caused acute disease. These undesired outcomes suggest that *in vitro* replication rate alone is not a reliable indicator of a virus’ clinical fitness^10^,^11^. We recently showed that an epidemiologically fitter strain of dengue virus (DENV) that emerged during the 1994 dengue outbreak in Puerto Rico paradoxically replicated more slowly than the virus it displaced^12^. The fitter virus “sacrificed” its genomic RNA to form more subgenomic RNA, which downregulated type-I interferon expression by binding a RIG-I signalling pathway intermediate. The reduced type-I interferon signalling likely then allowed DENV to spread to more susceptible cells, eventually reaching higher viraemia levels for more efficient vector-borne transmission^12^. Our findings thus indicate that viral replication rate is not invariably correlated with viral fitness in an epidemiological or clinical setting.

To gain clinically informed insights into the molecular determinants of attenuation, we examined a LAV candidate that caused acute illness in phase I clinical trials. The strains in the tetravalent dengue LAV developed at Mahidol University were selected for passaging primarily based on the small plaque phenotype; DENV in cells surrounding small plaques were purified and expanded after every five passages^4^. Other empirically defined markers used to determine attenuation were temperature sensitivity and reduced neurovirulence in suckling mice^4^,^5^. Despite this uniform approach, the vaccine candidates had contrasting clinical outcomes.

DENV serotype 3 (DENV-3) vaccine strain DENV-3 PGMK30FRhL3 (hereafter referred to as PGMK30) was generated by passaging wild-type DENV-3 16562 thirty times in primary green monkey kidney cells (PGMK) and three times in primary foetal rhesus lung (FRhL) cells. In a human safety trial, however, all volunteers who received tetravalent LAV formulation containing PGMK30 developed symptoms and signs consistent with acute dengue, with detectable DENV-3 viraemia^10^. Following the disappointing clinical trial results, this DENV-3 vaccine strain was further passaged in Vero cells using the same Mahidol University protocol to yield the Vero-derived Vaccine (VDV3) strain, which again met the same phenotypic criteria for attenuation previously used. When VDV3 was administered to healthy volunteers, it again caused disease in recipients^13^,^14^. In contrast, the DENV-2 vaccine strain, PDK53, which was generated from wild-type virus (16681) after 53 passages in primary dog kidney (PDK) cells was clinically safe and has since been used as a backbone to construct chimeric vaccines for the other DENV serotypes^15^,^16^. Although follow up investigations on PGMK30 found differences in host cell responses compared to wild-type DENV-3^13^, the underlying reasons for why PGMK30 caused acute illness in vaccine recipients despite meeting the empirical criteria for attenuation were never determined. Understanding the mechanisms that operate to yield divergent clinical outcomes despite the similar small plaque phenotype in these two LAV candidates could enable a more objective and accurate approach to selecting attenuated strains for development into LAVs.

## Results

### Attenuation status is not correlated with rate of cell-to-cell spread or genomic replication of DENV

**Figure 1:**
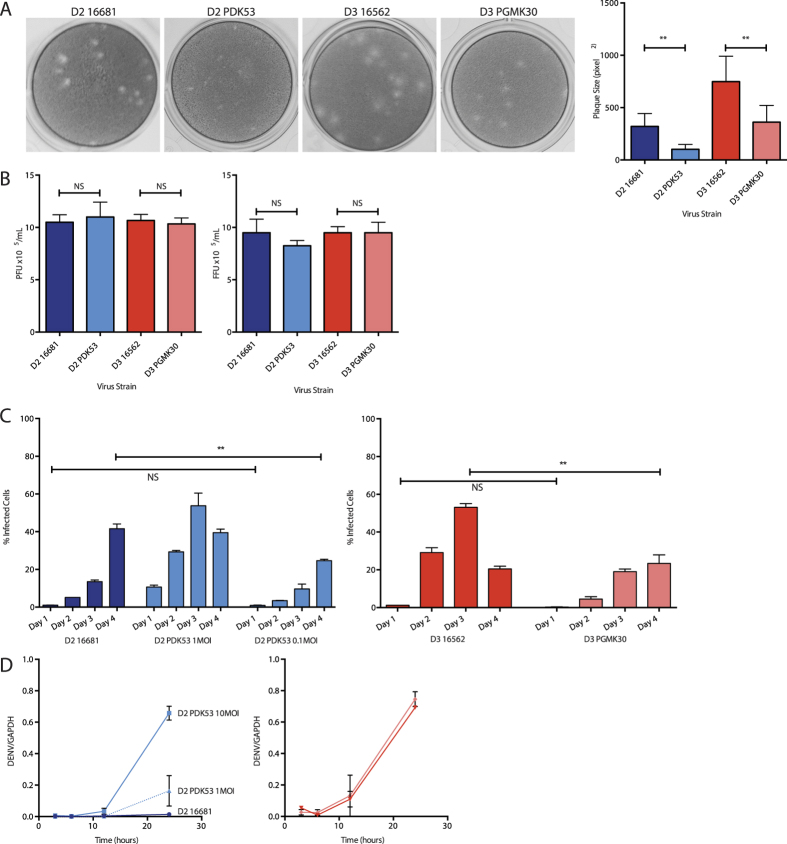
Properties of the vaccine and wild-type strains. (**A**) Representative photographs of viral plaques (left) and their corresponding plaque sizes in BHK-21 cells (right). (**B**) Plaque assay titres (left) and focus forming assay titres (right) of viral inoculum diluted to 10MOI (1 × 10^5^ per mL) for cell culture experiments, confirming that all strains have the same inoculum titres, and that the PFU and FFU titres corresponded to each other. (**C**) Cell-to-cell spread of virus in HuH-7 cells as measured by flow cytometry. At 24 hpi, 1MOI of PDK53 infected 10% of cells, approximately 10-fold more than 1MOI of 16681 (1%). Proportion of cells infected with PDK53 (0.1MOI) was similar to those infected with 16681 (1MOI) at 24 hpi although the latter overtook the former by day 4 of infection (left). In contrast, PGMK30 did not spread as rapidly as 16562 (right). (**D**) Viral RNA replication in HuH-7 cells as measured by qPCR in the first 24 hours of infection. At the earliest time point, 3 hpi, 10MOI of PDK53 produced significantly more viral RNA than 10MOI of 16681. Viral RNA levels were balanced with 16681 (10MOI) when PDK53 was infected at 1MOI. When starting levels of viral RNA are balanced, PDK53 replicated more rapidly than 16681 in the first 24 hours of infection (left). PGMK30 and 16562 replicated at the same rate (right).

We first confirmed the small plaque phenotype displayed by the attenuated PDK53 and PGMK30 strains, and their wild-type counterparts, on BHK-21 cells (Fig. 1A). The back-titrations of the viral inoculum (1 × 10^5^ pfu/mL) used in our experiments were verified using plaque as well as focus-forming assays to ensure that the viral titres were not underestimated due to the small plaque size. Both plaque and focus-forming unit assays produced consistent findings (Fig. 1B). Full genome sequencing of all four virus strains (Accession numbers in Supplementary Table 1) showed that passage of these viruses in our laboratory resulted in a variant with one amino acid substitution in the NS4A protein (N22D) of 61.33% of PDK53. This mutation is not known to be associated with a change in viral fitness. Importantly, the genome sequence of our 16681 strain exactly matched previous reports, while the three mutations in PDK53 previously shown to be critical for the attenuated state of PDK53^5^ were all preserved.

To measure the *in vitro* replication rate and cell-to-cell spread of the viruses, HuH-7 cells were infected with each of the DENV-2 or DENV-3 strains. At 24 hours post-infection (hpi), flow cytometry showed that PDK53 paradoxically infected a significantly higher proportion of cells (30%) than wild-type 16681 (5%). The proportion of 16681-infected cells at 24 hpi was instead equivalent to PDK53 infection at 10-fold lower MOI (Fig. 1C, left) although the proportion of 16681-infected cells overtook that for PDK53 at 96 hpi (42% versus 39%) (Fig. 1C). Conversely, the proportion of 16562-infected cells was significantly greater at 24, 48 and 72 hpi compared to PGMK30, with the former achieving infection percentages of 1%, 27% and 53% whilst the latter obtained 0.3%, 5% and 19%, respectively (Fig. 1C, right).

The RNA replication rate of these viruses was also measured using quantitative RT-PCR (qPCR)^17^. Consistent with the flow cytometry findings, PDK53 at 2 hpi had significantly greater RNA than 16681 when both viruses were inoculated onto Huh-7 cells at 10 MOI (Fig. 1D, left). Compared to PDK53 infection at 1 MOI, 16681 RNA levels at 10 MOI showed similar intracellular viral RNA initially, although by 24 hpi, PDK53 showed higher RNA levels than 16681 (Fig. 1D). In contrast, the RNA levels of the two DENV-3 strains were similar to each other in the first 24 hours of infection (Fig. 1D).

**Figure 2:**
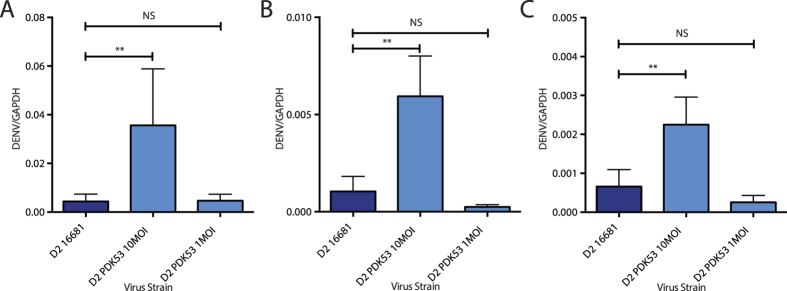
Viral RNA 2 hpi measured by qPCR of D2 infections. (**A**) DENV-2 RNA in Huh-7 cells treated with pronase to remove bound extracellular virions. Uptake of 10MOI PDK53 was approximately 10-fold greater than equivalent MOI of 16681. (**B**) DENV strains opsonised with humanised 4G2 monoclonal antibody infected THP-1 cells via Fc-receptor mediated uptake. 4G2 binds the fusion loop which is only exposed in mature virions. Uptake of 10MOI of PDK53 was 10-fold greater than 10MOI of 16681. (**C**) DENV-2 RNA in Raji cells expressing the DC-SIGN receptor. Uptake of PDK53 (10MOI) was 10-fold greater than equivalent MOI of 16681. All data points were calculated from at least 4 biological replicates; Error bars indicate standard deviation. P-values were calculated using a two-tailed t-test; *indicates p < 0.05 and **indicates p < 0.01 respectively.

**Figure 3:**
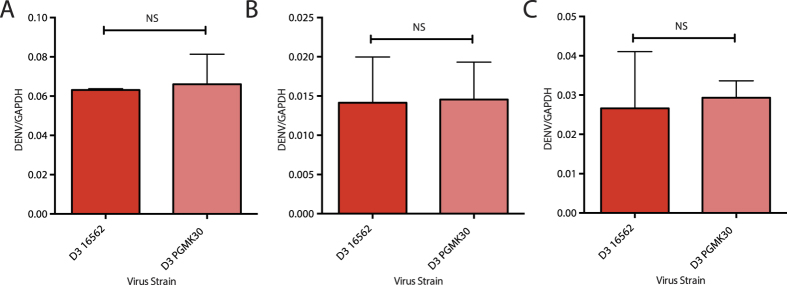
Viral RNA 2 hpi measured by qPCR of DENV-3 infections. (**A**) DENV-3 RNA in pronase-treated Huh-7 cells. (**B**) DENV-3 RNA in THP-1 cells infected via antibody-dependent pathway. (**C**) DENV-3 RNA in Raji cells expressing the DC-SIGN receptor. In all cases, the amount 16562 and PGMK30 uptake by cells were similar. All data points were calculated from at least 4 biological replicates; Error bars indicate standard deviation. P-values were calculated using a two-tailed t-test; *indicates p < 0.05 and **indicates p < 0.01 respectively.

To rule out virions bound to the cell membrane but not internalized as a potential source of this difference in viral RNA, we treated HuH-7 cells with 1 mg/mL pronase, a non-specific mixture of proteases able to cleave off surface-bound virions without causing cell lysis^18^. At 2 hpi and after pronase treatment, the viral RNA levels for 16681 at 10 MOI infection were equivalent to PDK53 at 1 but not 10 MOI (Fig. 2A). To verify that this was not unique to Huh-7 cells, we infected Raji cells stably expressing the dendritic cell-specific intercellular adhesion molecule-3-grabbing non-integrin (DC-SIGN) receptor, previously shown to facilitate DENV entry^19^,^20^. A similar trend to that observed in Huh-7 cells was obtained at 2 hpi (Fig. 2B). To ensure that our findings were not influenced by possible differences in the proportion of mature and immature virions, we infected THP-1 cells with PDK53 opsonized with enhancing concentrations of humanized 4G2 monoclonal antibody (mAb). 4G2 binds the highly conserved fusion loop of the E protein – only available for binding in mature virions – and standardizes viral entry through activating Fc receptors; infection rates of THP-1 cells without opsonising antibody is minimal^21^,^22^. Infection through this pathway produced similar trends in viral RNA levels to Huh-7 at 2 hpi (Fig. 2C). Conversely, in these same assays, we observed no differences in uptake between 16562 and PGMK30 (Fig. 3A–C).

Collectively, these findings indicate that the different levels of 16681 and PDK53 viral RNA post-inoculation is independent of cell type or receptor used to initiate entry. Furthermore, the higher than expected level of cytoplasmic RNA and proportion of infected cells for PDK53 suggest that plaque formation and the resultant plaque size are not determined by viral replication rate alone. Instead, host cell factors may regulate cell-to-cell spread: playing a critical role in the DENV-2 strains, and a much lesser one in DENV-3.

### PDK53 but not PGMK30 induces a robust antiviral response upon infection, limiting its *in vivo* spread

To compare the host response to the wild-type viruses and their respective small plaque-sized derivatives, we used an unbiased whole genome microarray to study changes in host gene expression. To ensure that the amount of cytoplasmic viral RNA is similar between 16681 and PDK53, we included infection with PDK53 at 1 and 10 MOI and compared the former to 16681 inoculated at 10 MOI (Fig. 2A). Infection with 16562 and PGMK30 was done at 10 MOI.

**Figure 4:**
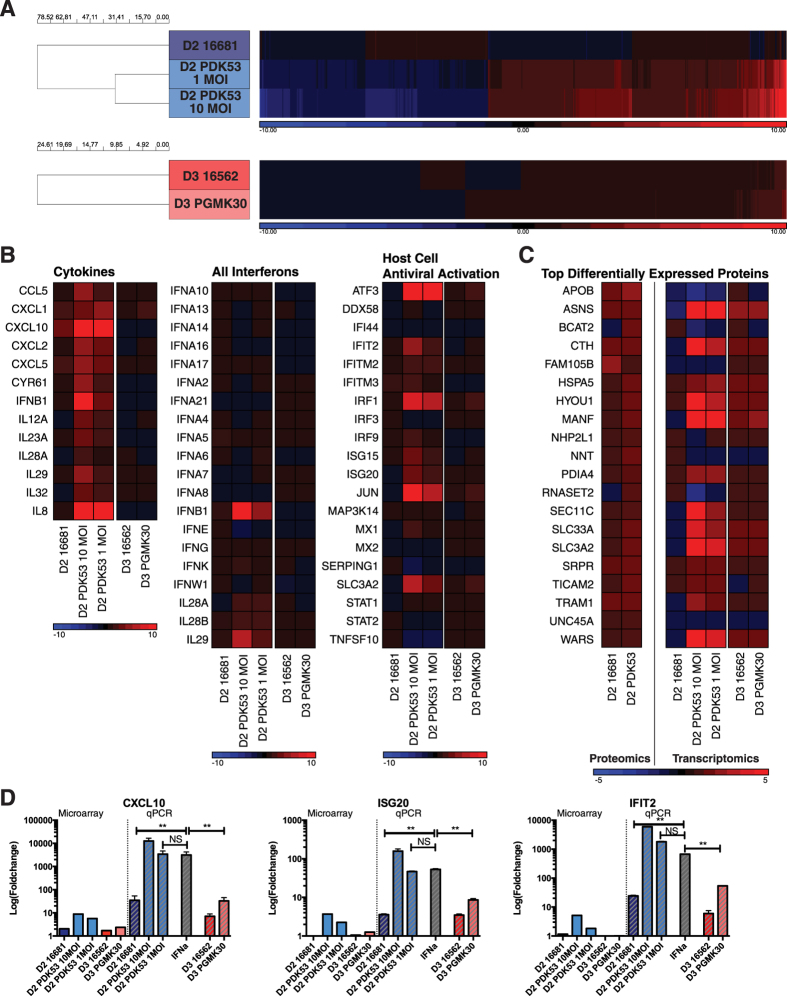
Host cell responses to infection with different DENV strains. (**A**) Unbiased whole genome microarray hits of significant transcriptomic changes in infected HuH-7 cells (>2-fold change relative to uninfected cells, corrected p-value <0.05) showing how only PDK53, whether at 1MOI or 10MOI, induced significant changes in gene transcription relative to uninfected cells. Cells infected by PGMK30 and both wild-type strains at 10MOI remained largely unperturbed by infection. Upregulated genes are depicted in red, and downregulated genes in blue. (**B**) Transcription changes in HuH-7 cells detected by microarray of selected genes, grouped by antiviral function. Only PDK53 consistently upregulated antiviral gene expression. Few changes were observed in PGMK30 relative to 16562 infection. (**C**) Translational differences (left heat map) and their corresponding microarray transcript levels (right heat map) of the most differentially enriched proteins in HuH-7 cells infected with either 16681 or PDK53. (**D**) mRNA levels of selected ISGs measured by qPCR of infected primary human monocytes (right side of graph), alongside their corresponding microarray transcript values of infected Huh-7 cells (left side of graph). Error bars indicate standard deviation. P-values were calculated using a two-tailed t-test; *indicates p < 0.05 and **indicates p < 0.01 respectively.

Interestingly, there were relatively few differentially expressed genes upon 16681 infection compared to uninfected control. In contrast, PDK53 infection resulted in significant changes in gene expression at both MOIs (Fig. 4A, top). Few differences were also observed in cells infected with either DENV-3 strain (Fig. 4A, bottom). Pathway analysis of genes upregulated by PDK53 infection reveals many with known antiviral functions, including cytokines, interferon-β (IFN-β), IL29 and interferon-stimulated genes (ISGs) (Fig. 4B). Because both DENV-3 strains minimally perturbed gene expression, we performed proteomic analysis only on DENV-2-infected cells. The results indicate increased expression of endoplasmic reticulum (ER) stress proteins, indicating that PDK53 infection resulted in increased transcriptional and translational activities in host cells (Fig. 4C).

The observed induction of antiviral response in HuH-7 cells by PDK53 but not PGMK30 or the wild-type viruses could be replicated in primary human monocytes. Infection of primary monocytes with PDK53 at both 1 and 10 MOI resulted in greater levels of CXCL10, ISG20 and IFIT2 mRNA levels compared to 10 MOI of 16681 (Fig. 4D). These ISGs are known to be important for the control of DENV replication in infection^23^. Infection with either 16562 or PGMK30 did not significantly induce the expression of these ISGs (Fig. 4D).

Thus far, our results suggest that PGMK30, despite its slow growth rate, remains able to evade antiviral activation unlike PDK53. To test if these antiviral responses played a role in controlling the spread of PDK53 *in vitro*, we used siRNAs to knock down signalling intermediates of their activation – MAVS, TRIF, and IRF3 – in HuH-7 cells. Treated cells were then infected with the four viruses as before. We found that only IRF3 knockdown increased the spread of PDK53 relative to control siRNA treatment (Supplementary Fig. 1). MAVS and TRIF knockdown did not affect the proportion of cells infected by PDK53; and the other three viruses were unaffected by any of the siRNA treatments. These findings suggest that of the four viruses, only PDK53 is constrained in spreading by antiviral responses; additionally, PDK53 appears capable of triggering more than one cellular pattern recognition receptor as MAVS or TRIF knockdown alone is not sufficient to affect its spread.

**Figure 5:**
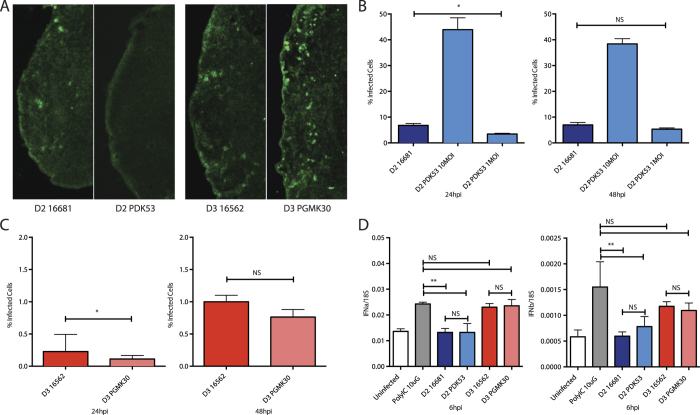
*In vivo* spread of DENV and adaptation to canine cells. (**A**) Immunofluorescence of DENV in popliteal lymph nodes 24 hours after inoculation of virus into the footpads of C57BL6 mice. 1 × 10^5^ pfu of DENV was injected subcutaneously into the footpads of immunocompetent C57BL/6 mice. The draining popliteal lymph nodes were dissected 24 hpi then stained for viral dsRNA (green). (**B**) Percentage of Madin-Darby Canine Kidney (MDCK) cells infected with DENV-2 as measured by FACS at 24 hpi (left) and 48 hpi (right). At 10MOI, PDK53 infected significantly more MDCK cells (44%) than 10MOI of 16681 (6%). At 1MOI, PDK53 infected fewer cells at 24 hpi (4%), but caught up with 16681 (6%) at 48 hpi. (**C**) MDCK cells infected DENV-3 at 24 hpi (left) and 48 hpi (right). Both DENV-3 strains infected approximately 1% of cells at both time points. (**D**) IFNα (left) and IFNβ (right) transcript levels in infected MDCK cells. PDK53, like 16681, did not significantly upregulate interferon expression, unlike infection with either DENV-3 strains or treatment with poly I:C.

The *in vitro* control of viral spread by the antiviral response could also have implications on the ability of PDK53 to spread *in vivo*, and thereby cause viraemia. To test how these viruses spread *in vivo*, we used an immunocompetent mouse model previously described^24^,^25^. The footpads of C57BL/6 mice were inoculated with one of the 4 viruses. After 24 hours, the draining popliteal lymph nodes were then dissected and stained for DENV. Confocal microscopy analysis found that PGMK30 was able to spread to the draining lymph nodes, just as well as wild-type 16562 and 16681. In contrast, PDK53 produced weak signals of infection in the draining lymph nodes (Fig. 5A).

The inability of PDK53 to evade antiviral activation in humans may be due to its adaptation to canine cells. Both 16681 and PDK53 infected Madin-Darby Canine Kidney (MDCK) cells, a dog kidney cell line (>5% infected cells at 24 hpi, and an increase in the proportion of infected cells on the second day of infection) (Fig. 5B) whereas 16562 or PGMK30 only yielded negligible levels of infection (<1% at both 24 hpi and 48 hpi) as measured by flow cytometry (Fig. 5C). Unlike infection in human cells, PDK53 induced IFN-α and-β expression at levels similar to 16681 in MDCK cells and is significantly lower than cells stimulated with the synthetic dsRNA analogue, poly I:C. In contrast, both 16562 and PGMK30 induced greater IFN-α and-β expression at 6 hpi (Fig. 5D).

### Innate immune responses controls the plaque size of PDK53 but not PGMK30

The observed differences in antiviral induction and growth rates between the DENV-2 and DENV-3 strains suggest that two fundamentally different mechanisms could be regulating the process of viral spread between PDK53 and PGMK30. PDK53 spread appears regulated by antiviral responses, while PGMK30 is merely slower at spreading to other cells. Thus far, these findings have been considered in HuH-7 cells, primary monocytes and in mice. Given that vaccine strain development is based primarily on plaque selection and purification, we examined if these two different mechanisms operate to determine viral plaque formation.

**Figure 6:**
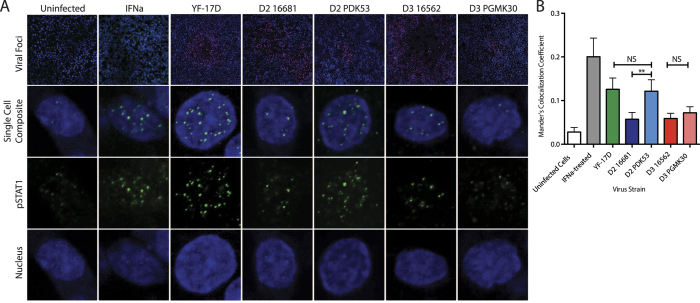
STAT1 activation in BHK-21 cells. (**A**) Representative images of confocal microscopy images of cells that were uninfected and untreated (negative control), interferon-treated (positive control), surrounding YF-17D or DENV focus of infection. Top row: 10X low power magnification view of viral foci, showing cell nuclei (blue), YF-17D or DENV (red), and pSTAT1 (green); Second row: Single cell nucleus composite image of an uninfected cell near focus of infection; Third and fourth rows: Single-channel images of the same cell showing pSTAT1 or cell nucleus, respectively. (**B**) Mander’s co-localisation coefficient measurements for nuclear-translocated pSTAT1 measured in uninfected cells in the periphery of viral foci. Levels of nuclear translocated pSTAT1 were similar for PDK53 and YF-17D, both of which were significantly higher compared to 16681, 16562 and PGMK30. P-values were calculated using a two-tailed t-test; **indicates p < 0.01.

To test whether the plaque size of PDK53 was limited by antiviral responses, we conducted an immunofluorescence-based focus-forming assay on BHK-21 cells, a cell line commonly used for plaque assays. Using confocal microscopy, we measured nuclear translocation of phosphorylated STAT1 (pSTAT1) in uninfected BHK-21 cells surrounding foci of viral infection (Fig. 6A). Increased levels of nuclear-translocated pSTAT1 were observed in IFN-treated or yellow fever vaccine (YF-17D)-infected cells that served as positive controls, relative to the uninfected negative controls (Fig. 6B). Likewise, significantly increased amounts of nuclear-translocated pSTAT1 were also observed in cells surrounding foci of PDK53 infection. In contrast, PGMK30, along with both wild-type viruses, did not elevate levels of nuclear-translocated pSTAT1 in the periphery of viral foci (Fig. 6B).

**Figure 7:**
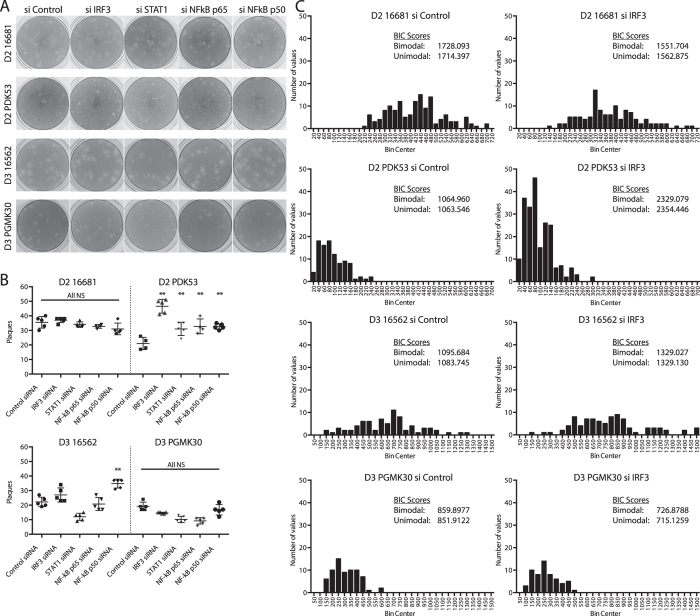
Effects of siRNA knockdown of antiviral activators on plaque size phenotype. (**A**) Photographs of representative plaque assays for each of the DENV strains in cells transfected with either scrambled siRNA or siRNA that targed IRF3, STAT1 or NF-kB subunits. (**B**) Plaque counts (per well of a 24-well plate) for each of the DENV strains in cells transfected with either scrambled siRNA or siRNA that targed IRF3, STAT1 or NF-kB subunits. Significant increase in pfu was observed in PDK53 when inoculated onto cells transfected with IRF3, STAT1 or NF-kB knockdown. Significant increase in 16562 pfu was observed with NF-kB p50 knockdown. The other viruses and treatments showed no significant change. P-values were calculated using a two-tailed t-test; ** indicates p < 0.01. (**C**) Plaque size distributions of the four virus strains for control and IRF3 knockdown, along with corresponding Bayesian Information Criterion (BIC) scores for unimodal or bimodal distribution. Lower BIC scores indicate better fit. All four control siRNA treatments yielded unimodal plaque size distributions. With IRF3 knockdown, PDK53 and 16681 plaques better fit a bimodal distribution, although the differences in plaque sizes were more distinct in PDK53. 16562 was neither more unimodal nor bimodal, whilst PGMK30 better fit a unimodal distribution.

If the plaque size of PDK53 were limited by the antiviral response, then interfering with its activation – by silencing IRF3, STAT1 and NF-kB – would be expected to change the plaque characteristics of PDK53, but not PGMK30 or the wild-type viruses. Representative photographs of the resulting plaques in BHK21 cells, which are commonly used for plaque assays, are shown in Fig. 7A. Firstly, when infected onto the plaque assay wells at the same inoculum of 30 pfu per well, PDK53 yielded more plaques when IRF3, STAT1 or NF-kB were silenced, indicating that many PDK53 infections do not normally form visible plaques or foci of infection due to antiviral activation. In contrast, silencing any of these genes did not result in significant change in the plaque count for PGMK30 or either wild-type DENV (Fig. 7B).

Secondly, the plaque phenotype also appeared to change with some siRNA treatments. The increase in plaque counts and plaque sizes due to siRNA treatment could not be discriminated by flow cytometry as both would result in an increased proportion of infected cells. Calculations of mean plaque size were similarly confounded by the appearance of new plaques of varying sizes, and hence could not be directly compared.

We thus analysed whether IRF3, STAT1 or NF-kB knockdowns caused plaque sizes to become unimodally or bimodally distributed, with bimodal distribution indicating that some enlarged plaques occurred in a background of new, smaller plaques. We mathematically fit the respective plaque size measurements into both unimodal and bimodal distributions, and then calculated Bayesian information criterion (BIC; the smaller the more plausible) scores for both types of distributions. When cells were treated with control siRNA, the resulting plaques better fitted a unimodal distribution except for PDK53, which was equivocal. BIC scores suggest that with IRF3 knockdown, PDK53 plaques fell into a bimodal distribution while PGMK30 plaques were unimodally distributed. Of the two wild-type strains, 16681 appeared to be bimodal, while 16562 was equivocal (Fig. 7C). The plaque size distribution of PDK53 in STAT1 or NF-kB p50 subunit knockdown cells, and 16681 in STAT1 knockdown cells also favoured bimodal distribution. Both DENV-3 strains yielded plaque size distributions that were either unimodal or equivocal; none showed clear bimodal distribution (Supplementary Fig. 2).

## Discussion

The goal of LAV development is to derive a viral strain with reduced virological fitness that causes asymptomatic acute infection; the immune response to this infection protects against subsequent wild-type infection. The process of generating attenuated strains has remained fundamentally unchanged since pioneering work by Sabin and others in developing the oral polio vaccine^26^. Unfortunately, the empirical nature of serial passaging in cells means that successful attenuation is a hit-or-miss affair. For viruses without reliable preclinical predictors of attenuation, testing for vaccine effectiveness can only happen in phase I clinical trial – posing problems of both safety and cost. Understanding the molecular basis behind viral attenuation is thus a critical step towards improving LAV development.

Collectively, our findings indicate that serial passaging in canine cells coupled with small plaque selection has yielded a canine-adapted viral strain that has lost its ability to evade human antiviral defences. Conversely, as monkeys are evolutionarily closer to humans, viruses adapted to PGMK likely retained their ability to suppress the human antiviral response. Consequently, selecting for small plaques could have yielded a strain that replicates slowly but which remains virulent in humans.

Previous studies on successful LAVs have identified key genomic correlates of protective immune responses^13^,^14^,^27^,^28^,^29^,^30^. Of particular interest is the prior work done comparing responses in human myeloid dendritic cells infected with 16562, the Vero-derived successor of PGMK30 (VDV3), or the yellow fever-dengue chimeric vaccines (CYD1-4). These studies found differences in the transcription of key ISGs, pro-inflammatory cytokines, and dendritic cell activation markers – with the strongest activation by CYD vaccines, while minimal immune activation was observed with the reactogenic VDV3 strain^14^. Despite differences in infection protocols and the viruses used, as well as their smaller subset of genes tested by cDNA macro-array, the broad conclusions agree with our findings, that successful live-attenuated vaccines trigger larger-scale and more robust changes in whole-genome transcription compared to unattenuated virus strains^27^,^28^.

The transcriptional changes induced by DENV-2 strains were also previously studied, in which 16681 was found to have induced greater transcriptomic changes in infected primary human monocytes compared to their vaccine strain^31^. These findings contrast with ours although the inoculum was based on MOI rather than internalised viral RNA nor were the proportion of infected cells ascertained to be equal between 16681 and PDK53. Moreover, as with the DENV-3 studies, the authors used a cDNA macro-array restricted to 268 pre-selected genes and were therefore not able to sample changes in the transcriptome globally upon infection with either DENV-2 strain.

Besides detailing the host response to infection, our findings further expand on the literature by identifying determinants of plaque size, which is often the primary selection criterion for attenuation. Our study thus adds a layer of information unaddressed in prior work, which is critical for the field to avoid repeating costly failures. Our data collectively indicate that there are at least two distinct molecular determinants of the small plaque phenotype in dengue LAV strains. PDK53 grows rapidly immediately upon infection but its cell-to-cell spread is curtailed by its inability to evade antiviral responses in primate or rodent cells. In human recipients, these responses limit the spread of PDK53 while triggering adaptive immunity. In contrast, the small plaque phenotype of PGMK30 is due to its slow growth rate, without compromising its ability to evade immune detection. It thus spreads more effectively *in vivo*, possibly with a longer incubation period before disease onset.

Collectively, these findings suggest that selection for strains that have adapted to evading antiviral activation in non-primate species and are thus unable to evade antiviral responses in humans is a key safety feature. Indeed, the yellow fever YF-17D vaccine was passaged through mouse brain followed by chicken embryos^32^. Measles and mumps vaccine were developed in eggs^6^,^7^. Rubella vaccine is an exception to this trend as it was passaged in human diploid fibroblast cells but adapted to replication at 30 °C, compromising replication at fever temperatures^33^. Conversely, other attempts to passage viruses in primate or human cells, such as the Towne^11^ or AD169^34^ strains for human cytomegalovirus, and even the VDV3 vaccine strain^14^, ultimately yielded reactogenic viruses.

Another interesting finding is the higher than expected viral content of PDK53 at equal MOIs with 16681. We conclude that the higher viral titer comes from additional productive infections, which are only revealed in the plaque assay after antiviral activation is impaired. Because the additional plaques fall into a bimodal distribution with IRF3 knockdown, we also conclude that the original plaques must have also increased in size once the constraint on viral spread is impaired. We are unable to determine if our NS4A variant subpopulation contributed to these findings. However, since our PDK53 genome sequence otherwise matches previously published sequences, including the genome position 5270 A/T variant^35^, and contain no reversions to wild-type, the additional mutation is more likely to increase rather than decrease attenuation. We thus believe that the presence of this amino acid variant does not affect our overall interpretation of live vaccine safety. There is therefore a possibility that the higher than expected PDK53 inoculum could contribute to immunogenicity although this interpretation cannot be fully ascertained in the background of the NS4A variant. As for the wild-type 16681 strain, although its plaque sizes also split into a bimodal distribution with IRF3 knockdown, there are no additional plaques that result from siRNA treatment and so this likely represents a broadening of the range of plaque sizes, rather than any ability to trigger antiviral responses.

In conclusion, our findings indicate that at least two different molecular mechanisms govern the plaque phenotype and that determining which mechanism operates to cause small plaque size may be a more accurate way to select future live-attenuated vaccine strains for clinical development.

## Materials and Methods

### Cells, Viruses, and Reagents

HuH-7 and Raji cells stably expressing DC-SIGN (gift from Timothy Burgess^20^,^36^) were cultured in Dulbecco’s Modified Essential Medium (DMEM) while THP-1 and BHK21 cells were cultured in RPMI Medium 1640 (Gibco) supplemented with 10% foetal calf serum. Primary human monocytes were derived from blood obtained from the principal investigator using a protocol previously described^37^. DENV strains of the original Mahidol stocks (DENV-2 16681, DENV-2 PDK53, DENV-3 16562 and DENV-3 PGMK30FRhL3) were obtained from Dr Claire Huang (Centers for Disease Control and Prevention, USA) and were passaged three times in C6/36 cells in our laboratory. To achieve sufficiently high titres for subsequent experiments, supernatant of PDK53 cultures were concentrated by high-speed centrifugation and reconstituted in 1/100 of its original culture volume. Viral genome sequences were uploaded to GenBank with the accession numbers listed in Supplementary Table 1. Yellow fever YF-17D was commercially obtained (Sanofi Pasteur).

### Transcriptomic Analysis

HuH-7 cells seeded in a confluent monolayer were infected with 10MOI of DENV-2 16681 or the DENV-3 strains; or 1MOI and 10MOI of DENV-2 PDK53. At 24 hpi, total cellular RNA was extracted using the RNEasy Mini kit (Qiagen) and then analysed on the Illumina HumanHT-12 v4 Expression Beadchip (Illumina). Results were analysed using Partek Genomics Suite v6.6 ©2014 (Partek). Pathway analysis was done using Gene Set Enrichment Analysis (Broad Institute, USA)^38^,^39^.

### Flow cytometry

Proportion of infected cells was assessed by flow cytometry. Cells were fixed and permeabilised with 3% paraformaldehyde and 0.1% saponin (Sigma-Aldrich) respectively, stained for DENV envelope (E) protein with mouse monoclonal 4G2 antibody (ATCC) and AlexaFluor488 anti-mouse secondary antibody (Invitrogen); then analysed on a BD FACSCanto II (BD Bioscience). Each data point represents a minimum of 5000 events and three biological replicates.

### Quantification of Viral RNA Uptake and Replication

Total cellular RNA was extracted using the RNEasy Mini kit (Qiagen), and complementary DNA synthesised using the iScript cDNA Synthesis kit (Bio-Rad). To measure total viral RNA, quantitative real-time PCR was done with iQ SYBR Green Mastermix (Bio-Rad) using a primer targeting a highly conserved region of the 3′ UTR common to all four serotypes of dengue as previously reported^40^; inter-sample normalization was done using GAPDH as a control. Primer sequences are listed in Supplementary Table 2. Pronase (Roche) was used at a concentration of 1 mg/mL and incubated with infected cells for five minutes on ice, before washing with ice cold PBS. Total cellular RNA was then extracted from the cell pellets in the manner described above.

### Animal models

C57BL/6 mice were purchased from InVivos (Singapore) and maintained in the Duke-NUS Vivarium for the duration of the experiment. The SingHealth Institutional Animal Care and Use Committee approved all mouse experiments and experiments were carried out in accordance with approved guidelines. Mice were infected by injecting 100 μL of 1 × 10^6^ pfu/mL DENV subcutaneously into the hind footpads. Draining popliteal lymph nodes were collected 24 h post-infection, snap frozen in O.C.T. Compound (Tissue-Tek), then cryosectioned (10 μm thick sections). Sections were fixed with acetone at 4 °C then stained using J2 anti-dsRNA antibody (English and Scientific Consulting) and FITC-conjugated anti-mouse antibody (Jackson ImmunoResearch). Slides were imaged by confocal microscopy. Images were prepared for publication using ImageJ (National Institutes of Health, USA)^41^.

### Plaque and Focus-Forming Assays

Culture supernatant, or virus samples, underwent serial 10-fold dilutions, then incubation on a monolayer of BHK-21 cells for 1 h at 37 °C. Samples were removed and replaced with 0.8% carboxymethylcellulose in RPMI supplemented with 3% foetal calf serum. Cultures for plaque assay were incubated for 6 days at 37 °C, then fixed with 20% formaldehyde and stained with 1% crystal violet (Sigma-Aldrich) to visualize plaques. Cultures for focus forming assay were incubated for 3 days at 37 °C, then fixed with 3% paraformaldehyde, permeabilised with 0.1% saponin, then stained with mouse 4G2 monoclonal antibody and anti-mouse horseradish peroxidase antibody. Viral foci were stained with 3-3′-diaminobenzidine (DAB Chromogen) (Dako) and enumerated visually.

### Confocal Imaging for Viral Foci

BHK-21 cells in a confluent monolayer on glass coverslips were infected with 30 pfu of virus and incubated for 72 hours at 37 °C. IFNα (Abcam) was used as a positive control. The cells were fixed and permeabilised with 3% paraformaldehyde and 0.1% saponin, then stained with anti-phospho-STAT1 (Y701) rabbit polyclonal IgG (R&D Systems), J2 anti-dsRNA mouse antibody, DAPI, AlexaFluor488 anti-rabbit, and AlexaFluor594 anti-mouse secondary antibodies (Invitrogen). Coverslips were affixed onto glass slides using Mowiol 4–88 (Sigma-Aldrich) and imaged using an LSM 710 confocal microscope (Carl Zeiss).

### Immune pathway knockdown and plaque phenotype studies

BHK-21 cells seeded in a confluent monolayer were treated with the specified hamster-specific siRNA at 100 nM final concentration complexed with Dharmafect 4 (GE Healthcare) for 48 hours. siRNA sequences used are listed in Supplementary Table 3. Following siRNA knockdown, each well was infected with 30 pfu of virus, and then incubated as for the plaque assay. Resultant plaques were photographed using an EOS 5D digital SLR camera (Canon) at a fixed distance. Plaque sizes were measured in pixels using ImageJ.

## Additional Information

**How to cite this article**: Goh, K. C. M. *et al*. Molecular determinants of plaque size as an indicator of dengue virus attenuation. *Sci. Rep.* **6**, 26100; doi: 10.1038/srep26100 (2016).

## Supplementary Material

Supplementary Information
