# Supplementary material for: Molecular determinants of plaque size as an indicator of dengue virus attenuation
Source: Sci Rep. 2016 May 17;6:26100. doi: 10.1038/srep26100 (PMC4868997; doi:10.1038/srep26100)

Molecular determinants of plaque size as an indicator of dengue virus attenuation

Kenneth Choon Meng Goh[1], Choon Kit Tang[1], Diana Catherine Norton[2], Esther Shuyi Gan[1], Hwee Cheng Tan[1], Bo Sun[2], Ayesa Syenina[1], Amjad Yousuf[3], Xin Mei Ong[1], Uma Sangumathi Kamaraj[1], Yin Bun Cheung[1], Duane J Gubler[1], Andrew Davidson[3], Ashley Lauren St John[1], October Michael Sessions[1], Eng Eong Ooi[1*]

[1]Duke-NUS Medical School, Singapore 169857, Singapore

[2]Duke University School of Medicine, Durham, North Carolina 27710, USA

[3]University of Bristol, Bristol BS8 1TD, UK

Supplementary Information

Molecular determinants of plaque size as an indicator of dengue virus attenuation – Supplementary Information

Supplementary Table 1: Dengue Genome Sequences

**Virus Strain**	**GenBank Accession Number**
DENV-2 16681	KU725663
DENV-2 PDK53	KU725664
DENV-3 16562	KU725665
DENV-3 PGMK30FRhL3	KU725666

Supplementary Table 2: PCR Primer Sequences

**Gene Target**	**Primer Sequence**
DENV LYL 3'UTR	Forward: TTGAGTAAACYRTGCTGCCTGTAGCTC<br>Reverse: GAGACAGCAGGATCTCTGGTCTYTC
GAPDH (Human)	Forward: GAGTCAACGGATTTGGTCGT<br>Reverse: TTGATTTTGGAGGGATCTCG
CXCL10 (Human)	Forward: GGTGAGAAGAGATGTCTGAATCC<br>Reverse: GTCCATCCTTGGAAGCACTGCA
ISG20 (Human)	Forward: ACACGTCCACTGACAGGCTGTT<br>Reverse: ATCTTCCACCGAGCTGTGTCCA
IFIT2 (Human)	Forward: GAAGAGGAAGATTTCTGAAG<br>Reverse: CATTTTAGTTGCCGTAGG
IFNa (Canine)	Forward: GCTCTTGTGACCACTACACCA<br>Reverse: AAGACCTTCTGGGTCATCACG
IFNb (Canine)	Forward: GGATGGAATGAGACCACTGTCG<br>Reverse: ACGTCCTCCAGGATTATCTCCA

Supplementary Table 3: BHK21 siRNA Sequences (*Mesocricetus auratus*)

Gene Target	siRNA Sequence
IRF3	Sense: GGAACAAUGGGAGUUCGAAdTdT<br>Antisense: UUCGAACUCCCAUUGUUCCdTdT
STAT1	Sense: CCGUUUCCAUGACCUCCUUdTdT<br>Antisense: AAGGAGGUCAUGGAAACGGdTdT
NF-kB p65	Sense: GCAUCCAGACCAACAAUAAdTdT<br>Antisense: UUAUUGUUGGUCUGGAUGCdTdT
NF-kB p50	Sense: CCUCAUGUUCACCGCCUUUdTdT<br>Antisense: AAAGGCGGUGAACAUGAGGdTdT

Molecular determinants of plaque size as an indicator of dengue virus attenuation – Supplementary Information

Supplementary Figure 1:

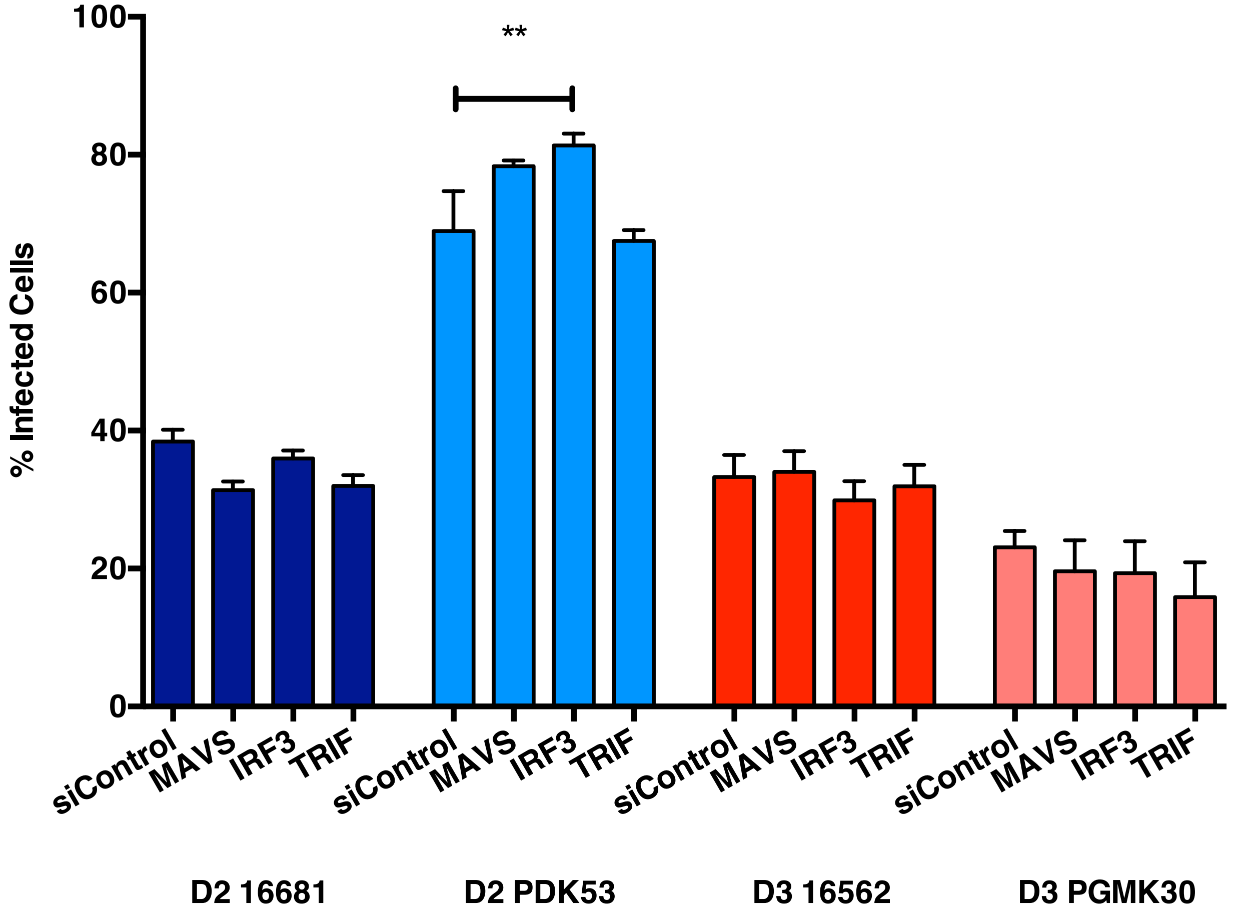

Supplementary Figure 1: Percentage of infected HuH-7 cells after siRNA measured by flow cytometry. There is a modest increase in the amount of cells infected by PDK53, especially with IRF3 knockdown; the other viruses are not affected.

Molecular determinants of plaque size as an indicator of dengue virus attenuation – Supplementary Information

Supplementary Figure 2:

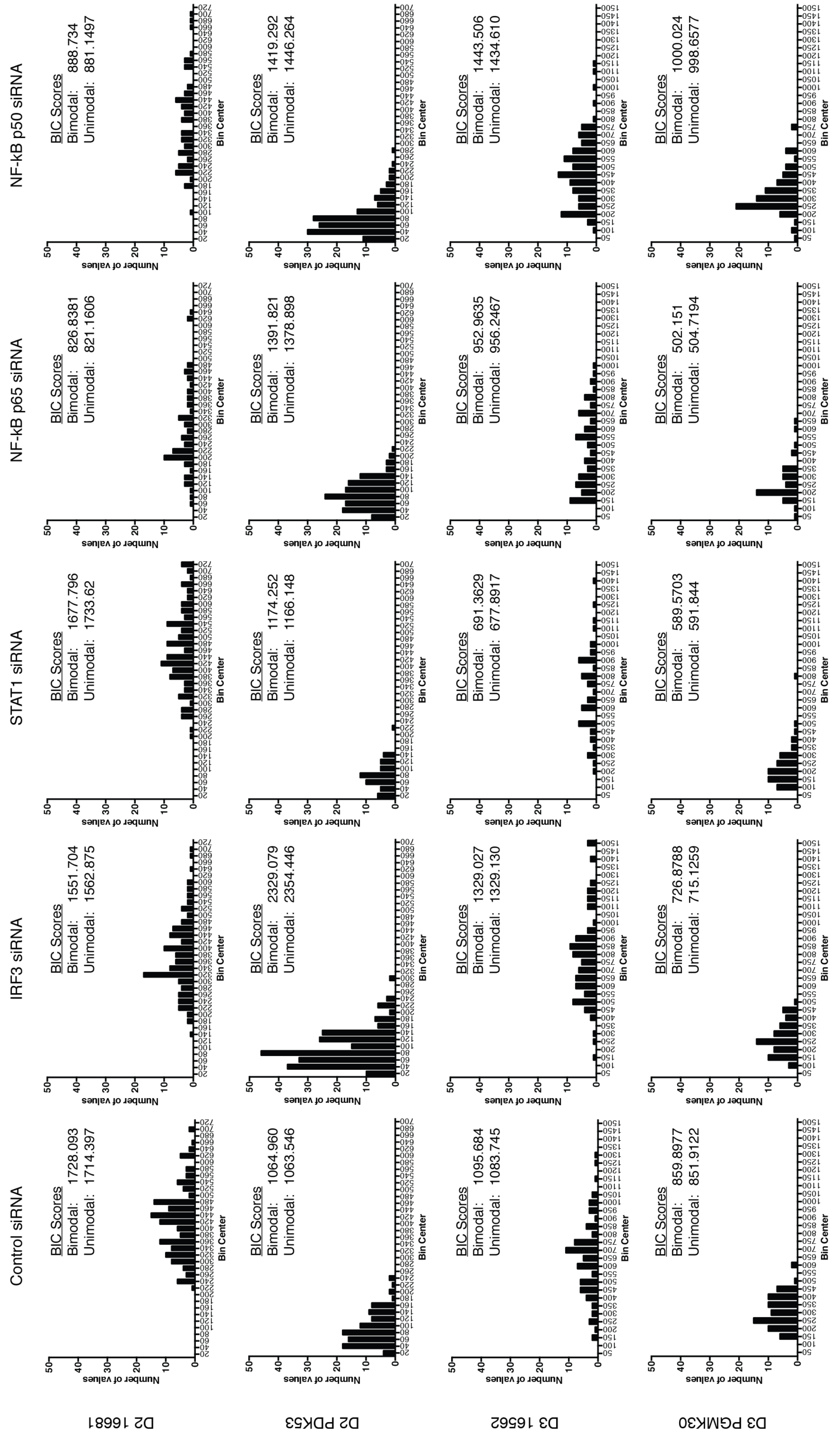

Supplement: Supplementary Information [file srep26100-s1.pdf]
